# Red yeast rice-induced severe rhabdomyolysis complicated by acute kidney injury and respiratory failure: a case report

**DOI:** 10.3389/fmed.2025.1634047

**Published:** 2025-08-20

**Authors:** Pengmin Zhou, Yucai Hong, Huabo Cai, Xiaoyu Zhou, Shunpeng He, Haotian Zhou, Jie Yang, Pengpeng Chen, Boming Xia, Xiong Lei, Suibi Yang, Zhongheng Zhang

**Affiliations:** Department of Emergency Medicine, Sir Run Run Shaw Hospital, Zhejiang University School of Medicine, Hangzhou, China

**Keywords:** red yeast rice, rhabdomyolysis, acute kidney injury, respiratory failure, statin-associated myopathy

## Abstract

Red yeast rice (RYR), a commonly used supplement with statin-like properties, is generally considered safe but may cause severe adverse effects such as rhabdomyolysis. We report a rare case of severe RYR-induced rhabdomyolysis complicated by acute kidney injury (AKI) and respiratory failure, with diaphragmatic dysfunction as a key contributing factor. A 78-year-old woman developed progressive proximal muscle weakness, dyspnea, and tea-colored urine after taking RYR (2 g/day) for 1 month. She rapidly progressed to respiratory failure requiring intubation and mechanical ventilation. Laboratory tests showed a peak creatine kinase (CK) of 112,985 U/L, serum myoglobin (>3,000 μg/L), and AKI. Bedside ultrasound demonstrated diaphragmatic dysfunction, while electromyography (EMG) revealed preserved nerve conduction. Myositis-specific and paraneoplastic antibody panels were negative. She received continuous renal replacement therapy (CRRT), plasma exchange (PE), hemoperfusion (HP), and supportive care. During hospitalization, she developed deep vein thrombosis (DVT), pneumonia, and ileus, all of which resolved with treatment. At discharge, she had been weaned from mechanical ventilation and had partially recovered renal and muscle function. At follow-up, she was stable, ambulating, and performing daily functions without symptom recurrence. Although her initial presentation mimicked immune-mediated necrotizing myopathy (IMNM), the absence of myositis-specific antibodies and clinical improvement without immunosuppressive therapy supported a diagnosis of toxic rhabdomyolysis. This case highlights the importance of recognizing supplement-related toxicity and initiating timely organ-targeted supportive care. This appears to be the first reported case of RYR-induced rhabdomyolysis complicated by both AKI and respiratory failure from diaphragmatic dysfunction.

## Introduction

Red yeast rice (RYR) is a traditional Chinese nutraceutical obtained by fermenting white rice with the mold *Monascus purpureus*. It has gained global popularity as a dietary supplement for cholesterol reduction, primarily due to its content of monacolin K—a naturally occurring statin analog chemically identical to lovastatin (1). Although. commonly marketed as a “natural” and safer alternative to prescription statins, RYR shares similar pharmacodynamic properties and, consequently, a comparable profile of adverse effects (1, 2)

Among these effects, rhabdomyolysis is a serious and potentially life-threatening complication, characterized by the breakdown of skeletal muscle and the subsequent release of intracellular contents—including creatine kinase (CK) and myoglobin—into the bloodstream (1, 3). While statin-induced rhabdomyolysis has been extensively described, reports of RYR-associated cases remain limited, and severe presentations involving multiorgan dysfunction are exceedingly rare (1).

Here, we present a rare and severe case of RYR-induced rhabdomyolysis in an elderly woman, uniquely complicated by acute kidney injury (AKI) and ventilatory failure secondary to diaphragmatic dysfunction. The clinical presentation mimicked statin-induced immune-mediated necrotizing myopathy (IMNM) in its early clinical course; however, the absence of myositis-specific antibodies and the patient's marked clinical improvement without immunosuppressive therapy supported a diagnosis of toxic rhabdomyolysis.

This appears to be the first documented case of RYR-induced rhabdomyolysis complicated by both AKI and ventilatory failure. This report highlights the potential systemic risks of over-the-counter supplements like RYR and emphasizes the need for clinical awareness.

## Case presentation

### History of present illness

A 78-year-old woman presented with a 9-day history of low back pain and progressive lower limb weakness, accompanied by worsening dyspnea over the past 3 days. The patient's symptoms began with acute-onset low back pain and leg fatigue, and she rapidly became unable to walk or sit unassisted. She subsequently developed slurred speech, chewing difficulty, and profuse sweating. At the referring hospital, 1 day before transfer to our ICU, she became somnolent due to carbon dioxide retention and required endotracheal intubation and mechanical ventilation. Arterial blood gas analysis performed at the referring hospital revealed a PaCO_2_ of 80 mmHg at the time of intubation.

### Past medical history

The patient had a longstanding history of hypertension for over 10 years, managed with oral amlodipine, and type 2 diabetes mellitus for ~6 months, which was managed with diet control alone. Her home supplements included Coenzyme Q10, Omega-3, and *Ganoderma lucidum* spore powder. Six months prior to the current admission, she was hospitalized for hyperglycemia management, during which routine tumor marker screening revealed elevated carcinoembryonic antigen (19.72 ng/ml) and carbohydrate antigen 72-4 (300 U/ml). However, subsequent abdominal imaging and endoscopic evaluations did not reveal any evidence of malignancy. Two months before admission, she underwent elective surgical resection of an ovarian cyst. Histopathological analysis confirmed the lesion to be benign. Notably, she began taking RYR (2 g daily) 1 month before symptom onset. She denied the use of any prescription statins during this period.

### Physical examination

Upon admission to the Intensive Care Unit (ICU), the patient was intubated and sedated, with stable vital signs. After cessation of sedation, she was fully alert and able to follow commands, although full orientation could not be assessed due to intubation. Neurological examination revealed symmetric proximal muscle weakness, graded as 2/5 in the upper limbs and 1/5 in the lower limbs according to the Medical Research Council (MRC) scale, while distal muscle strength was preserved at 4/5. Deep tendon reflexes were absent, and Babinski signs were negative bilaterally. Pupils were equal in size and reactive to light. Cardiopulmonary examinations were unremarkable. Abdominal examination revealed a soft, non-tender abdomen with normal bowel sounds. Tea-colored urine was noted through the indwelling Foley catheter.

### Laboratory and imaging examinations

On admission, laboratory tests revealed markedly elevated CK (30,454 U/L), serum myoglobin (>3,000 μg/L) and creatinine (167 μmol/L), consistent with severe rhabdomyolysis and acute kidney injury. Additional findings included significantly increased levels of lactate dehydrogenase (2,096 IU/L), alanine aminotransferase (ALT, 641 U/L), and aspartate aminotransferase (AST, 749 U/L). Her complete blood count on admission showed leukocytosis (white blood cell count, 17.1 × 10^9^/L), with a hemoglobin level of 134 g/L and a platelet count of 239 × 10^9^/L. Acute phase reactants were mildly elevated, with a C-reactive protein level of 11.0 mg/L and a procalcitonin level of 0.08 ng/ml. Urinalysis revealed both hematuria and proteinuria.

A comprehensive neuromuscular workup was performed to exclude other etiologies. Head computed tomography (CT) scans were normal. Lumbar puncture revealed clear cerebrospinal fluid (CSF) with no albuminocytologic dissociation. Electromyography (EMG) demonstrated mildly prolonged F-M latencies and reduced F-wave persistence, but motor and sensory nerve conduction studies were otherwise unremarkable, arguing against a primary neurogenic process.

To investigate for an autoimmune cause, comprehensive myositis-specific and paraneoplastic autoantibody panels were tested and found to be negative. These included myositis-specific antibodies (e.g., anti-HMGCR, anti-SRP, anti-Mi-2) and neuronal antibodies (e.g., anti-Hu, anti-Yo, anti-Ri). Further diagnostic investigations, such as muscle MRI or biopsy, were also deferred at this stage, given that MRI was unfeasible on a ventilated patient and biopsy results would not be timely enough to guide acute management.

To identify the cause of respiratory failure, a chest CT scan was performed and was normal. Subsequently, bedside diaphragm ultrasound performed during tidal breathing with pressure support of 5 cmH_2_O, focused on the right hemidiaphragm for optimal acoustic window and data quality, showing an excursion of 0.91 cm, end-expiratory thickness of 0.14 cm, end-inspiratory thickness of 0.18 cm, and a thickening fraction (TFdi) of 28.5%—findings consistent with right diaphragmatic dysfunction. A comprehensive assessment of global diaphragmatic function and other accessory respiratory muscles was not performed due to technical limitations.

### Diagnosis

The patient was diagnosed with severe rhabdomyolysis complicated by AKI and respiratory failure, likely induced by RYR intake. A detailed differential diagnosis is discussed below.

### Treatment and clinical course

**Figure 1:**
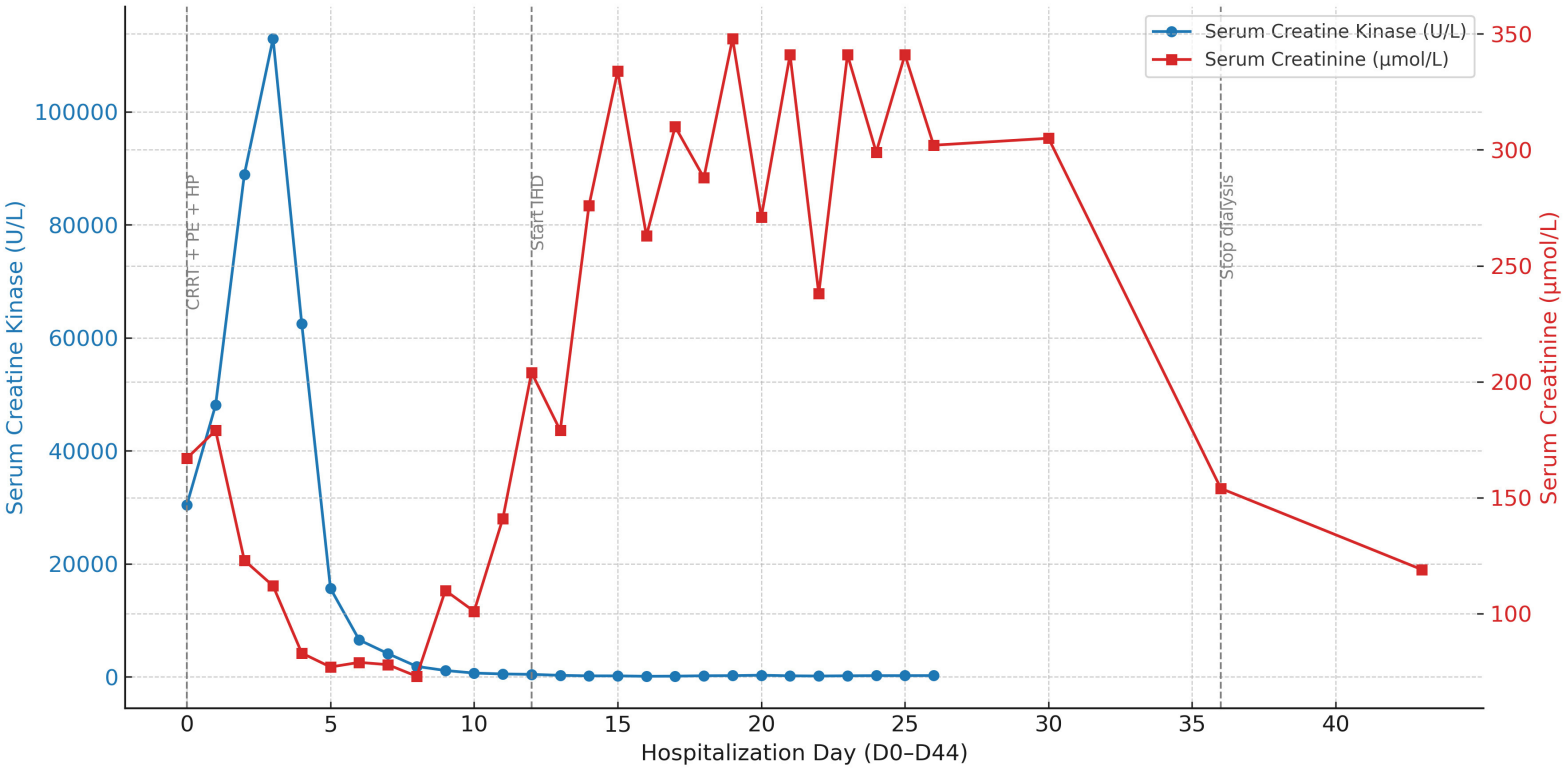
Temporal trends of serum creatine kinase and creatinine during hospitalization. This figure depicts the 45-day trajectory of serum creatine kinase (CK) and serum creatinine (Cr) levels in a patient with red yeast rice (RYR)-induced rhabdomyolysis, complicated by acute kidney injury and respiratory failure. CK (blue solid line with circular markers, left *Y*-axis) and Cr (red solid line with square markers, right *Y*-axis) are plotted to reflect muscle injury and renal function, respectively. Gray vertical dashed lines denote three major therapeutic stages: (1) early intensive therapy, including continuous renal replacement therapy (CRRT, D0–11), plasma exchange (PE, D1, 3, and 5), and hemoperfusion (HP, D2); (2) initiation of intermittent hemodialysis (IHD) on D12; and (3) termination of dialysis on D35. The dual *Y*-axis design facilitates simultaneous visualization of muscular and renal biomarker dynamics in relation to therapeutic interventions. Clinically, these trends illustrate that the resolution of severe rhabdomyolysis (indicated by the early decline in CK) preceded the delayed recovery of consequent acute kidney injury, which required a prolonged period of dialysis before renal function improved (indicated by the decline in Cr after D35). CK, creatine kinase; Cr, creatinine; CRRT, continuous renal replacement therapy; PE, plasma exchange; HP, hemoperfusion; IHD, intermittent hemodialysis.

The initial step in management was the immediate discontinuation of RYR. On D0 (counted from ICU admission; see Table 1), aggressive intravenous hydration and urine alkalinization were initiated to support renal protection. However, due to persistent oliguria, worsening metabolic acidosis, and rising creatinine levels, continuous renal replacement therapy (CRRT) was promptly started. Given the limited efficacy of CRRT in clearing myoglobin, and in light of the early diagnostic ambiguity between toxic and immune-mediated processes, three sessions of therapeutic plasma exchange (PE) were performed for potential immunomodulation and enhanced myoglobin clearance. Additionally, one session of hemoperfusion (HP) was undertaken to further assist in myoglobin removal, although its role remains investigational. As shown in Figure 1, the patient's CK level peaked at 112,985 U/L on D3. With a sustained decline in CK levels and gradual clinical improvement, CRRT was subsequently transitioned to intermittent hemodialysis (IHD) on D12, administered three times per week. Trends in serum CK and creatinine levels throughout the hospitalization, along with the corresponding therapeutic interventions, are presented in Figure 1.

**Table 1:** Clinical timeline of the patient.

**Hospital day**	**Event**
D-9	Back pain and leg weakness onset
D-3	Worsening dyspnea
D-1	Somnolence due to hypercapnia; intubated
D0	ICU admission; CRRT started
D1	Plasma exchange × 1
D2	Hemoperfusion × 1; lower limb DVT
D3	Plasma Exchange × 1
D4	CK decreased; rehab initiated
D5	Plasma Exchange × 1
D8	VAP with fever; treated with antibiotics
D11	CRRT discontinued
D12	Hemodialysis started; paralytic ileus noted
D16	Tracheostomy; G3 dysphagia on Kubota test
D19	Ventilator weaned
D20	Afebrile; infection improved
D22	Paralytic ileus resolved
D28	Tracheostomy capped
D35	Renal recovery; dialysis stopped
D42	Swallowing adequate; oral intake resumed
D44	Stable discharge

CRRT, continuous renal replacement therapy; DVT, deep vein thrombosis; CK, creatine kinase; VAP, ventilator-associated pneumonia; G3, grade 3 dysphagia based on the Kubota water swallow test. D0 indicates the day of ICU admission; D–x represents days prior to ICU admission.

In parallel with ongoing renal support, early rehabilitation training was initiated on D4 to maintain muscle strength and support respiratory function. Due to persistent difficulty in weaning from mechanical ventilation, likely related to diaphragmatic dysfunction, a tracheostomy was performed on D16. Following tracheostomy, swallowing evaluation was performed for the first time, as reliable assessment had not been feasible during intubation. The Kubota water swallow test revealed grade 3 dysphagia. Accordingly, nasogastric feeding, which had been initiated during intubation, was continued following the tracheostomy.

As respiratory function continued to improve, the patient was successfully weaned from mechanical ventilation on D19. After further clinical stabilization, she was transferred to the rehabilitation unit on D25, where she participated in a comprehensive multidisciplinary program comprising physical therapy, occupational therapy, acupuncture, and physiotherapy, aimed at enhancing cardiopulmonary endurance and restoring both upper and lower limb muscle strength. On D35, serum creatinine levels had returned to near baseline, and hemodialysis was discontinued. The patient maintained adequate urine output and stable renal function thereafter. On D42, a videofluoroscopic swallowing study confirmed adequate swallowing function, and oral intake was safely resumed. The patient was discharged in stable condition on D44.

Her clinical course was also complicated by deep vein thrombosis (DVT), ventilator-associated pneumonia, and paralytic ileus, all of which resolved with targeted therapies and did not significantly delay her overall recovery. A timeline of key clinical events during the patient's hospital stay is summarized in Table 1.

### Outcome

After 45 days of hospitalization, the patient was discharged in a clinically stable condition, with spontaneous respiration, intact cognition, and hemodynamic stability. The tracheostomy tube was capped. Swallowing function improved (Kubota grade 2), and oral intake was resumed.

Muscle tone was normal [Modified Ashworth Scale (MAS) score 0]. Muscle strength was graded as 4+ proximally in the upper limbs, 3/5 in the lower limbs, and 5/5 distally, according to the MRC scale. The Modified Barthel Index was 30, reflecting a high level of functional dependency. She could not complete the 6-min walk test due to limited endurance. Cognitive function was intact, with a Mini-Mental State Examination score of 30. Serial assessments of muscle strength, swallowing ability, activities of daily living, and cognitive function are summarized in Table 2.

**Table 2:** Functional and neurological assessment.

**Parameter**	**D0**	**D4**	**D10**	**D24**	**D28**	**D44**
Upper limbs—proximal	2/5 (MRC)	2+/5	3/5	4/5	4+/5	4+/5
Upper limbs—distal	4/5	4/5	4/5	4+/5	4+/5	5/5
Lower limbs—proximal	1/5	2/5	2+/5	2+/5	2+/5	3/5
Lower limbs—distal	4/5	4/5	4/5	4/5	4/5	5/5
Deep tendon reflexes	Absent	NA	NA	NA	++	++
Muscle tone (MAS)	G0	G0	G0	G0	G0	G0
Kubota swallow test	NA	NA	NA	G3	G3	G2
Sitting/Standing balance	NA	NA	NA	NA	0/0	2/0
ADL (MBI)	NA	NA	NA	NA	10	30
6-min walk test	NA	NA	NA	NA	NC	NC
MMSE	NA	NA	NA	NA	28	30

MRC, Medical Research Council scale (used for muscle strength assessment, range 0–5); MAS, Modified Ashworth Scale; G, grade; G0, grade 0; G2, grade 2; G3, grade 3; NA, not assessed; NC, not completed; ADL, activities of daily living; MBI, Modified Barthel Index; MMSE, Mini-Mental State Examination; Absent, deep tendon reflexes not elicited; ++, normal deep tendon reflexes.

Muscle strength (rows 1–4) is graded using the MRC scale.

Sitting/Standing balance is presented as X/Y, where X = sitting balance score and Y = standing balance score.

Urine output was adequate, with stable creatinine and CK levels. Hemodialysis was discontinued. The patient was transferred to a local hospital for continued rehabilitation. Her recovery trajectory underscores the effectiveness of early organ-targeted supportive care and multidisciplinary rehabilitation in the management of severe toxic rhabdomyolysis.

### Follow-up

At ~3 months after symptom onset, a telephone follow-up was conducted. The patient had successfully undergone decannulation of the tracheostomy tube and was able to walk and engage in light outdoor activities such as strolling. She resumed basic daily activities independently. Renal function remained stable without recurrence of AKI, and serum creatinine levels continued to be within normal limits. No relapse of muscle weakness or respiratory symptoms was reported.

## Discussion

RYR, a fermented product rich in monacolin K—a compound chemically identical to lovastatin—is widely used as a lipid-lowering supplement. Though perceived as a “natural” alternative to prescription statins, RYR carries comparable pharmacological effects and potential for adverse reactions, including hepatotoxicity, myopathy, and in rare cases, severe rhabdomyolysis (1).

In the present case, the patient developed severe systemic manifestations ~1 month after initiating RYR supplementation, raising important diagnostic and therapeutic considerations. We herein discuss three key aspects of this case: differential diagnosis, systemic complications, and therapeutic challenges.

### Differential diagnosis: distinguishing myopathic from neurogenic weakness and excluding IMNM

The patient presented with acute symmetrical proximal muscle weakness and respiratory failure, prompting a systematic neuromuscular evaluation. Given the recent intake of RYR, a statin-like toxic effect was initially suspected. Nevertheless, because such presentations may arise from a broad spectrum of neuromuscular disorders, distinguishing between myopathic and neurogenic causes was essential.

Unlike the classic ascending, distal-to-proximal pattern of weakness in Guillain–Barré syndrome (GBS), our patient exhibited symmetrical proximal muscle weakness, a hallmark of myopathies (4). Moreover, while areflexia is common in GBS, it is not pathognomonic; critically ill patients may transiently lose reflexes due to severe myopathy or sedation. Lumbar puncture revealed normal CSF parameters without albuminocytologic dissociation, further lowering the likelihood of GBS (5). EMG demonstrated normal nerve conduction velocities and amplitudes, which were also inconsistent with a neurogenic process (4).

Laboratory findings strongly supported a myopathic process. While markedly elevated serum CK (peaked at 112,985 U/L) and myoglobin (>3,000 μg/L) were pathognomonic for severe myonecrosis, the transaminase profile was more complex. Initially, it followed a pattern suggestive of muscle injury, with a predominant rise in AST and preserved hepatic synthetic and excretory functions (6). However, the co-existing marked elevation of ALT pointed to a composite etiology, likely involving both direct release from myonecrosis and a concurrent hypoxic liver injury (6, 7). Collectively, the clinical presentation, electrophysiological studies, and laboratory data pointed toward a primary myopathic etiology.

Following exclusion of neurogenic causes, attention shifted to primary myopathies. Given the patient's RYR supplementation, which contains statin-like compounds, and the presence of respiratory involvement, IMNM was considered (8). IMNM typically presents with subacute proximal muscle weakness, sustained CK elevation, and positivity for anti-HMGCR or anti-SRP antibodies (9). Respiratory failure, a known complication of severe IMNM, is rarely seen in toxic rhabdomyolysis, making the clinical presentation particularly concerning. Although myositis-specific antibodies were negative, further evaluation with muscle biopsy or MRI is generally recommended in antibody-negative cases to confirm the diagnosis (10–12). However, MRI was not feasible due to mechanical ventilation, and muscle biopsy was expected to take more than 4 weeks for processing. Therefore, a stepwise approach was adopted: we prioritized clinical observation and supportive treatment, with the intent to reassess the need for further testing based on therapeutic response. The patient exhibited progressive clinical improvement and a steady decline in CK levels without immunosuppressive therapy. This favorable recovery trajectory was inconsistent with IMNM and instead supported a diagnosis of toxic rhabdomyolysis.

Other potential etiologies such as viral myositis or inherited metabolic myopathies were also considered, but deemed less likely due to the absence of a viral prodrome and the patient's advanced age of first presentation, respectively.

Thus, we clinically diagnosed RYR-induced toxic rhabdomyolysis based on the temporal association with supplement use, muscle involvement pattern, negative autoantibodies, and favorable response to supportive care. Consequently, further investigations such as muscle biopsy and MRI were deemed unnecessary.

### Systemic complications of RYR-induced rhabdomyolysis: AKI and respiratory failure

Rhabdomyolysis is characterized by extensive skeletal muscle breakdown, resulting in the release of intracellular components—such as myoglobin, CK, and electrolytes—into the circulation (13). In severe cases, this process may lead to systemic complications, including AKI, electrolyte imbalances, and, more rarely, respiratory failure (14–16).

In this case, two major complications developed.

First, AKI manifested early, with oliguria and a progressive rise in creatinine, necessitating CRRT, followed by IHD. This course was consistent with myoglobin-induced acute tubular injury (17).

Second, ventilatory pump failure, primarily driven by diaphragmatic dysfunction, manifested as severe hypercapnic (Type II) respiratory failure necessitating intubation. This conclusion was supported by objective data: the absence of pulmonary or cardiac causes on a normal chest CT scan and preserved cardiac function ruled out primary lung parenchymal diseases and cardiogenic pulmonary edema. Additionally, massive pulmonary embolism was considered unlikely given the patient's stable hemodynamics and the predominantly hypercapnic (Type II), rather than hypoxemic (Type I), nature of the respiratory failure. Primary neurological causes for ventilatory failure were also systematically excluded. A central nervous system origin was inconsistent with the patient's alertness and ability to follow commands when not sedated, a finding supported by a normal head CT scan. Peripheral neuropathies, such as Guillain–Barré Syndrome, were ruled out by the myopathic pattern of weakness, normal cerebrospinal fluid analysis, and normal nerve conduction studies (4, 5). Furthermore, bedside ultrasound of the right hemidiaphragm, a reliable indicator of overall diaphragmatic function in systemic myopathy, revealed significantly reduced excursion (0.91 cm; normal >1.34 cm) and thickening fraction (TFdi 28.5%; normal 30%−36%), findings consistent with diaphragmatic dysfunction (18). The patient's profound, symmetrical proximal limb weakness (MRC 1-2/5) provided clear clinical evidence of global myopathy, suggesting that accessory respiratory muscles (e.g., intercostals, scalenes), being skeletal muscles, were also likely affected by the same myotoxic process. The progression to overt respiratory failure demonstrated the decompensation of the entire respiratory muscle apparatus, as the weakened accessory muscles could not sustainably compensate for the failing diaphragm. The temporal correlation between massive CK elevation and respiratory failure, followed by respiratory recovery as CK levels declined, supports diaphragmatic dysfunction secondary to toxic rhabdomyolysis.

Our case shares key similarities and differences with other rare reports of severe rhabdomyolysis complicated by respiratory failure.

For instance, Gindre et al. described a case of cytomegalovirus (CMV)-associated rhabdomyolysis in an immunocompetent adult, complicated by respiratory failure due to generalized muscle weakness, with no pulmonary or cardiac pathology identified (19).

Additionally, Gentili et al. reported a pediatric case of carnitine palmitoyltransferase II (CPT II) deficiency in which acute respiratory failure was the first manifestation of rhabdomyolysis. The authors attributed this to severe muscle injury and hypoventilation, although specific diaphragmatic dysfunction was not confirmed (20).

In a population-based study, Devalaraju et al. (21) found that respiratory failure was a significant predictor of in-hospital mortality in patients with concurrent pancreatitis and rhabdomyolysis. While respiratory muscle function was not directly assessed, they hypothesized that damage to respiratory musculature might partly explain the increased mortality (21). A detailed comparison of these cases is presented in Table 3.

**Table 3:** Comparison of rhabdomyolysis cases with respiratory failure.

**Feature**	**Present case (This Study)**	**Gindre et al. (19)**	**Gentili et al. (20)**	**Devalaraju et al. (21)**
Study type	Case Report	Case Report	Case Report	NIS Database Study
Etiology	RYR toxicity	CMV infection	CPT II deficiency (triggered by fever and fasting)	Concurrent pancreatitis
Patient profile	78-year-old woman	32-year-old woman	4-year-old boy	5,421 patients
Peak CK (U/L)	112,985	177,000	320,000	Not reported
Key complications	Respiratory failure and AKI	Respiratory failure (no AKI)	Respiratory failure and AKI	Respiratory failure is a predictor of mortality
Mechanism of respiratory failure	Confirmed diaphragmatic dysfunction	Generalized muscle weakness	Severe muscle injury; diaphragmatic dysfunction not confirmed	Hypothesized to be respiratory muscle damage
Outcome	Partial recovery with residual weakness	Full recovery	Full recovery	High mortality in those with multi-organ failure; individual recovery not reported

RYR, red yeast rice; CMV, cytomegalovirus; CPT II, carnitine palmitoyltransferase II; AKI, acute kidney injury; NIS, nationwide inpatient sample.

However, none of these prior cases involved RYR as the precipitating factor. The 2025 EFSA safety assessment acknowledged severe adverse effects of RYR but did not document respiratory failure cases (1). Such severe systemic involvement following RYR use has, to our knowledge, not been previously reported.

Thus, this case expands the known spectrum of RYR toxicity, demonstrating that it may involve respiratory skeletal muscles and cause life-threatening ventilatory failure. Ongoing vigilance for multiorgan dysfunction is essential in patients with supplement-induced severe myopathies.

### Therapeutic challenge: myoglobin clearance and renal protection

Management of severe RYR-induced rhabdomyolysis presents significant therapeutic challenges, particularly in achieving effective myoglobin clearance and renal protection. Prompt discontinuation of the offending agent, aggressive intravenous hydration, and renal support remain the cornerstone of treatment (1).

In our patient, a multimodal approach was employed, including CRRT, plasma exchange, and hemoperfusion. CRRT was essential for maintaining fluid, electrolyte, and acid-base balance; however, its efficacy in eliminating myoglobin is limited by the molecule's intermediate size and protein-binding characteristics (22). Plasma exchange was initiated early due to diagnostic uncertainty, as IMNM and GBS had not been excluded at admission. It was considered for its potential immunomodulatory effects and possible myoglobin clearance, although supporting evidence remains limited (23). Hemoperfusion, used adjunctively in this case, is a promising yet investigational approach for enhancing myoglobin removal in severe rhabdomyolysis (22).

Despite these interventions, there is no standardized or highly effective method for targeted myoglobin clearance. The patient's recovery with supportive care highlights the effectiveness of early, organ-targeted management, even without specific myoglobin removal techniques. Nonetheless, the protracted renal recovery and dialysis dependence during hospitalization underscore the limitations of current therapies.

Future advances may focus on novel hemoadsorption devices, such as CytoSorb^®^ cartridges, which have shown potential for improving myoglobin clearance when initiated early (22). Furthermore, early biomarkers predicting severe AKI risk and individualized thresholds for initiating extracorporeal therapies could refine treatment algorithms. Prospective studies are urgently needed to establish evidence-based protocols for optimizing myoglobin clearance and improving renal outcomes in severe rhabdomyolysis.

### Limitations

Our assessment of respiratory muscle function, while indicative of diaphragmatic dysfunction, had certain limitations. First, the sonographic evaluation of the diaphragm, a primary muscle of respiration, was confined to the right hemidiaphragm. While this approach is justified by its technical feasibility and improved data quality compared to left-sided assessment, it precludes a complete evaluation of global diaphragmatic function and does not account for potential asymmetric involvement. Second, a specific sonographic assessment of the accessory inspiratory muscles (e.g., parasternal intercostals) was not performed. Such an evaluation could have provided additional quantitative data regarding the patient's respiratory workload and the degree of compensatory recruitment from these secondary muscle groups. Nevertheless, the systemic nature of rhabdomyolysis strongly implies global respiratory muscle involvement, consistent with the observed ventilatory pump failure.

### Patient perspective

During her prolonged and arduous hospitalization, the patient expressed significant regret over her use of the supplement, attributing her severe illness to its consumption. Her recovery was slow, which was a source of considerable anxiety, and she frequently voiced concerns about the extent of her long-term recovery and whether she would regain her independence. However, her determination throughout rehabilitation, culminating in the functional improvements documented at follow-up, ultimately tells a story of resilience in the face of a life-threatening, supplement-induced critical illness.

## Conclusion

We report a rare and severe case of RYR-induced toxic rhabdomyolysis complicated by AKI and respiratory failure secondary to diaphragmatic dysfunction, which, to our knowledge, is the first documented case of RYR-induced rhabdomyolysis complicated by both AKI and respiratory failure. The diagnostic challenge with IMNM was clarified by negative serology and rapid clinical improvement without immunosuppressive therapy. The patient recovered following early withdrawal of RYR and organ-targeted supportive care.

This case highlights the critical importance of early recognition of supplement-induced toxic myopathies, timely differentiation from autoimmune neuromuscular disorders, and prompt initiation of multidisciplinary supportive therapies.

Clinicians should remain highly vigilant for serious complications, even with seemingly “natural” over-the-counter products such as RYR, particularly in older adults with comorbidities.

Further research is needed to better delineate the clinical spectrum of RYR toxicity and to develop evidence-based strategies for myoglobin clearance and organ protection in rhabdomyolysis-associated AKI.

## Data Availability

The original contributions presented in the study are included in the article/supplementary material, further inquiries can be directed to the corresponding author.

## References

[1] Efsa Panel on Nutrition NF Food A Turck D Bohn T Camara M Castenmiller J. Scientific opinion on additional scientific data related to the safety of monacolins from red yeast rice submitted pursuant to article 8(4) of regulation (Ec) No 1925/2006. EFSA J. (2025) 23:e9276. 10.2903/j.efsa.2025.9276 40027377 PMC11868785

[2] Cicero AFG Fogacci F Stoian AP Toth PP. Red yeast rice for the improvement of lipid profiles in mild-to-moderate hypercholesterolemia: a narrative review. Nutrients. (2023) 15:2288. 10.3390/nu15102288 37242171 PMC10221652

[3] Wang YH Zhang SS Li HT Zhi HW Wu HY. Rhabdomyolysis-induced acute kidney injury after administration of a red yeast rice supplement: a case report. World J Clin Cases. (2023) 11:5547–53. 10.12998/wjcc.v11.i23.5547 37637685 PMC10450378

[4] Yuki N Hartung HP. Guillain-Barre syndrome. N Engl J Med. (2012) 366:2294–304. 10.1056/NEJMra1114525 22694000

[5] van Doorn PA Ruts L Jacobs BC. Clinical features, pathogenesis, and treatment of Guillain-Barre syndrome. Lancet Neurol. (2008) 7:939–50. 10.1016/S1474-4422(08)70215-1 18848313

[6] Weibrecht K Dayno M Darling C Bird SB. Liver aminotransferases are elevated with rhabdomyolysis in the absence of significant liver injury. J Med Toxicol. (2010) 6:294–300. 10.1007/s13181-010-0075-9 20407858 PMC3550495

[7] Fuhrmann V Kneidinger N Herkner H Heinz G Nikfardjam M Bojic A. Hypoxic hepatitis: underlying conditions and risk factors for mortality in critically ill patients. Intensive Care Med. (2009) 35:1397–405. 10.1007/s00134-009-1508-2 19506833

[8] Allenbach Y Benveniste O Stenzel W Boyer O. Immune-mediated necrotizing myopathy: clinical features and pathogenesis. Nat Rev Rheumatol. (2020) 16:689–701. 10.1038/s41584-020-00515-9 33093664

[9] Tiniakou E Christopher-Stine L. Immune-mediated necrotizing myopathy associated with statins: history and recent developments. Curr Opin Rheumatol. (2017) 29:604–11. 10.1097/BOR.0000000000000438 28857949

[10] De Visser M. Inflammatory myopathies: which diagnostic tools should we use? J Neurol Sci. (2023) 455:120916. 10.1016/j.jns.2023.120916

[11] Merlonghi G Antonini G Garibaldi M. Immune-mediated necrotizing myopathy (Imnm): a myopathological challenge. Autoimmun Rev. (2022) 21:102993. 10.1016/j.autrev.2021.102993 34798316

[12] Fionda L Lauletta A Leonardi L Perez JA Morino S Merlonghi G. Muscle Mri in immune-mediated necrotizing myopathy (Imnm): implications for clinical management and treatment strategies. J Neurol. (2023) 270:960–74. 10.1007/s00415-022-11447-7 36329184 PMC9886642

[13] Stahl K Rastelli E Schoser B. A systematic review on the definition of rhabdomyolysis. J Neurol. (2020) 267:877–82. 10.1007/s00415-019-09185-4 30617905

[14] Kodadek L Carmichael Ii SP Seshadri A Pathak A Hoth J Appelbaum R. Rhabdomyolysis: an American Association for the Surgery of trauma critical care committee clinical consensus document. Trauma Surg Acute Care Open. (2022) 7:e000836. 10.1136/tsaco-2021-000836 35136842 PMC8804685

[15] Chatzizisis YS Misirli G Hatzitolios AI Giannoglou GD. The syndrome of rhabdomyolysis: complications and treatment. Eur J Intern Med. (2008) 19:568–74. 10.1016/j.ejim.2007.06.037 19046720

[16] Hirohama D Shimizu T Hashimura K Yamaguchi M Takamori M Awatsu Y. Reversible respiratory failure due to rhabdomyolysis associated with cytomegalovirus infection. Intern Med. (2008) 47:1743–6. 10.2169/internalmedicine.47.1349 18827428

[17] Bosch X Poch E Grau JM. Rhabdomyolysis and acute kidney injury. N Engl J Med. (2009) 361:62–72. 10.1056/NEJMra0801327 19571284

[18] Zambon M Greco M Bocchino S Cabrini L Beccaria PF Zangrillo A. Assessment of diaphragmatic dysfunction in the critically ill patient with ultrasound: a systematic review. Intensive Care Med. (2017) 43:29–38. 10.1007/s00134-016-4524-z 27620292

[19] Gindre H Feasson L Auboyer C Cathebras P. Severe rhabdomyolysis associated with a primary cytomegalovirus infection in an immunocompetent patient. BMJ Case Rep. (2013) 2013:bcr2012008140. 10.1136/bcr-2012-008140 23413290 PMC3603949

[20] Gentili A Iannella E Masciopinto F Latrofa ME Giuntoli L Baroncini S. Rhabdomyolysis and respiratory failure: rare presentation of carnitine Palmityl-transferase Ii deficiency. Minerva Anestesiol. (2008) 74:205–8. 18414363

[21] Devalaraju SS Gundlapally MS Shaikh A Malangu B Ahlawat S. S3211 outcomes of patients admitted with concurrent pancreatitis and rhabdomyolysis: an Nis Database Study. Am J Gastroenterol. (2021) 116:S1322-S. 10.14309/01.ajg.0000786376.76569.f7

[22] Forni L Aucella F Bottari G Buttner S Cantaluppi V Fries D. Hemoadsorption therapy for myoglobin removal in rhabdomyolysis: consensus of the hemoadsorption in rhabdomyolysis task force. BMC Nephrol. (2024) 25:247. 10.1186/s12882-024-03679-8 39085790 PMC11293130

[23] Boparai S Lakra R Dhaliwal L Hansra RS Bhuiyan MAN Conrad SA. Therapeutic plasma exchange in severe rhabdomyolysis: a case-control study. Cureus. (2023) 15:e39748. 10.7759/cureus.39748 37398832 PMC10310893

